# Clinical outcomes of advanced non-small cell lung cancer patients harboring distinct subtypes of EGFR mutations and receiving first-line tyrosine kinase inhibitors: brain metastasis and de novo T790M matters

**DOI:** 10.1186/s12885-022-09245-5

**Published:** 2022-02-21

**Authors:** Ya Zeng, Tiantian Guo, Yue Zhou, Yang Zhao, Li Chu, Xiao Chu, Xi Yang, Jianjiao Ni, Zhengfei Zhu

**Affiliations:** 1 Department of Radiation Oncology, Fudan University Shanghai Cancer Center, Shanghai, 200032 China; 2 Department of Radiation Oncology, Shanghai Chest Hospital, Shanghai Jiao Tong University, Shanghai, 200030 China; 3 Department of Oncology, Shanghai Medical College, Fudan University, Shanghai, 200032 China; 4 Institute of Thoracic Oncology, Fudan University, Shanghai, 200032 China

**Keywords:** Non-small cell lung cancer, Uncommon EGFR mutations, Brain metastasis, de novo T790M, Clinical outcome

## Abstract

**Background:**

The clinical features, survival outcomes and patterns of treatment failure of advanced non-small cell lung cancer (NSCLC) patients harboring distinct subtypes of EGFR mutations and receiving first-line EGFR tyrosine kinases inhibitor (TKIs) are not fully understood.

**Methods:**

Consecutive metastatic EGFR-mutant NSCLC patients receiving first-line EGFR-TKIs from October 2010 to March 2020 were enrolled and classified into two main groups based on the EGFR mutation subtypes: common mutation (L858R or exon 19 deletion), uncommon mutation (other EGFR mutations).

**Results:**

Of the 1081 patients included, 74 (6.8%) harbored uncommon mutations. The baseline characteristics were generally balanced between the two groups, except that bone metastasis developed less frequently in patients with uncommon mutations (*p* = 0.02). No significant difference of survival outcomes was found between the two groups, except that among patients with baseline brain metastasis, the intracranial time to progression was significantly shorter in patients with uncommon mutations. Nine of the 17 patients with de novo T790M mutation received Osimertinib, whose overall survival tended to be longer than the remaining 8 patients without Osimertinib treatment (*p* = 0.08). The patterns of treatment failure were generally consistent between the two groups, except which patients with uncommon mutations had a higher risk developing progressive disease in the brain.

**Conclusion:**

First-line EGFR-TKIs seemed to be less effective in controlling and preventing brain metastasis in patients with uncommon EGFR mutations and Osimertinib was associated with promising efficacy in patients with de novo T790M mutation, which warranted further validation.

**Supplementary Information:**

The online version contains supplementary material available at 10.1186/s12885-022-09245-5.

## Introduction

Non-small cell lung cancer (NSCLC) is the leading cause of cancer-related death worldwide. And epidermal growth factor receptor (EGFR) mutations are frequently detected in advanced NSCLC. EGFR tyrosine kinase inhibitors (TKIs) are recommended as the first-line choice for patients with metastatic NSCLC harboring common sensitizing EGFR mutations (ie, L858R point mutation and exon 19 deletion) [1, 2]. Meanwhile, about 10% of the EGFR-mutant NSCLC patients harbor uncommon mutations (EGFR mutations except for L858R point mutation and exon 19 deletion) and the effectiveness of first-line EGFR-TKIs in these patients remain controversial [3–6]. Data from previous studies have shown that NSCLC patients harboring uncommon EGFR mutations do not have consistently favorable responses to EGFR-TKIs comparing to those with common mutations [3, 7–9]. The study performed by Shi et al. demonstrated that objective response rate (ORR) of patients harboring uncommon EGFR mutations who were treated with EGFR-TKIs was significantly inferior to that of patients harboring common mutations [10], which was consistent with the post-hoc analysis of the randomized NEJ002 clinical trial [9]. However, other studies reported conflicting results [11, 12]. The multicenter retrospective study from Japanese found that among metastatic NSCLC patients harboring uncommon EGFR mutations, the disease control rate and ORR of EGFR-TKIs treatment could reach 63.6 and 29.5%, respectively [13]. Moreover, based on data from a combined post-hoc analysis of LUX-Lung 2, LUX-Lung 3, and LUX-Lung 6, Afatinib was approved for the treatment of uncommon EGFR mutations and its efficacy was verified in several other studies [3, 4, 14]. Recently, Osimertinib demonstrated an ORR of 50% and median PFS of 8.2 months among NSCLC patients harboring uncommon EGFR mutations in the KCSG-LU15-09 study [15]. However, all of these studies mainly focused on the response rate and survival outcomes of EGFR-TKI treatment, while investigations about clinical features and patterns of treatment failure among these patients were seldom reported.

In addition, accumulating evidences suggest that different subtypes of EGFR mutations could have distinct response to the same EGFR-TKI and this may partially explain the conflicting results of EGFR-TKI treatment among patients harboring uncommon EGFR mutations, which consist of a heterogeneous subtypes of EGFR mutations [3, 14]. Among the various subtypes of uncommon EGFR mutations, there were 1% [12] to 40.9% [16] of patients harboring de novo T790M alone or concurrently with other sensitizing EGFR mutations before exposure to any kind of EGFR-TKIs. The clinical outcomes of these patients treated with different generations of EGFR-TKIs have been widely debated. Retrospective studies showed upset results of first-generation EGFR-TKIs for patients with de novo T790M, even coexisted with sensitizing EGFR mutations [17, 18]. The post-hoc analysis of LUX-Lung 2, LUX-Lung 3 and LUX-Lung 6 found that patients with de novo T790M could not derive significant benefit from Afatinib [3]. Report on patients with de novo T790M who were treated with Osimertinib was extremely rare. Zhang et al. reported that patients harboring coexistence of sensitizing EGFR mutations and de novo T790M could obtain a better survival benefit from third-generation EGFR-TKIs than that from first-generation EGFR-TKIs [19]. The efficacy of EGFR-TKIs treatment, especially third-generation EGFR-TKIs, among patients harboring de novo T790M warranted further research.

It is common for NSCLC patients to develop brain metastasis (BM) during their disease course and the optimal treatment strategies for EGFR-mutant NSCLC patients with BM are still under debate. Although EGFR-TKIs, especially Osimertinib, have been proved to introduce a potent response against BMs for patients harboring common EGFR mutations as shown in the FLAURA trial [20], the clinical outcomes of first-line EGFR-TKIs among patients harboring uncommon mutations and with BMs are largely unknown [21]. Hence, we performed a retrospective study examining the clinical features, survival outcomes and patterns of treatment failure in EGFR-mutant NSCLC patients receiving first-line EGFR-TKIs, with a special attention paid to the subgroups of patients with baseline brain metastasis and those with de novo T790M mutations.

## Materials and methods

### Patients

**Fig. 1:**
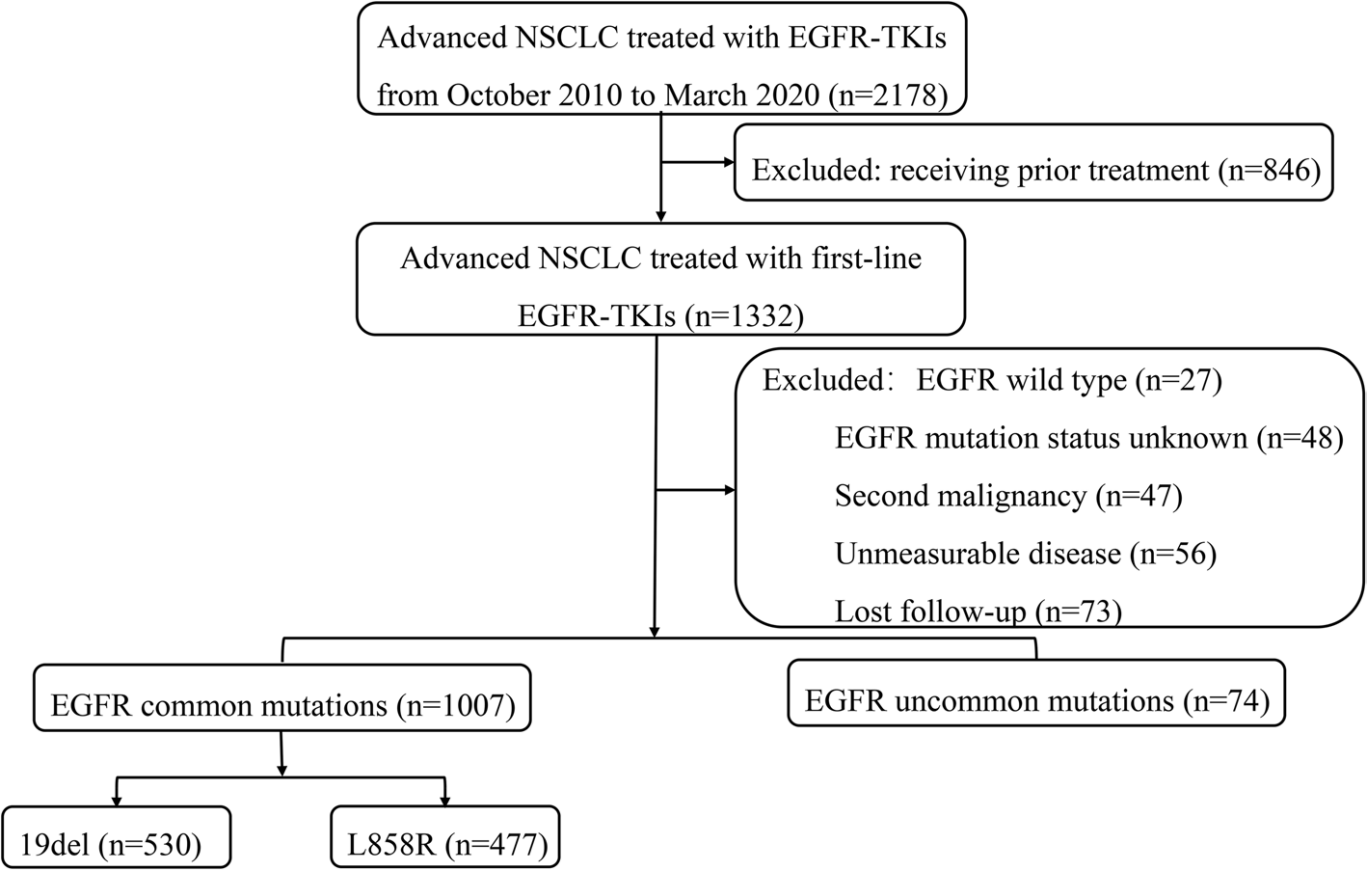
Patients’ selection flowchart

Patients with pathologically confirmed stage IV EGFR-mutant NSCLC and receiving first-line EGFR-TKIs in Fudan University Shanghai Cancer Center from October 2010 to March 2020 were retrospectively enrolled. The following patients’ demographic, clinical and molecular parameters were collected: age, gender, KPS score, mutation type, type of EGFR-TKIs and baseline metastatic sites (lung, bone, brain, liver, adrenal gland and distant lymph node). Patients who had measurable disease and regular follow-up were included. Detailed information about patients’ selection was displayed in Fig. 1. Of note, single L858R point mutation or exon 19 deletion (19del) was defined as common EGFR mutations, while other types of EGFR mutations were defined as uncommon mutations.

### Follow up

Follow ups were at the discretion of treating physicians and were generally performed every 2-3 months. Chest computed tomography (CT) scans and ultrasonography of abdominal and cervical regions were routinely performed. Meanwhile, brain magnetic resonance imaging (MRI) and bone scanning were regularly performed for those with baseline brain and bone metastases, respectively. Positron Emission Tomography (PET)-CT were not mandatory. Disease response was evaluated according to the Response Evaluation Criteria in Solid Tumor (RECIST) criteria (version 1.1).

### Statistical analysis

The oligo-metastatic status and oligo-progressive disease were defined strictly according to the previous studies [22, 23]. The OS and PFS was defined as the time from the date of diagnosis of advanced NSCLC to the date of death of any cause, and to the date of initial progressive disease (PD) or death of any cause, respectively. The intracranial time to progression (iTTP) was defined for patients with baseline BMs as the time from the date of diagnosis of advanced NSCLC to the date of intracranial PD. For those with initial extracranial PD only, iTTP was censored at the time of initial extracranial PD. OS, PFS and iTTP were estimated by Kaplan-Meier method. Log-rank tests were used to compare the survival curves. Propensity score matching (PSM) was used to adjust for the possible selection bias induced by the retrospective, nonrandomized design. *P* value less than 0.05 (two sided) was considered statistically significant in this study.

## Results

### Patient characteristics

**Fig. 2:**
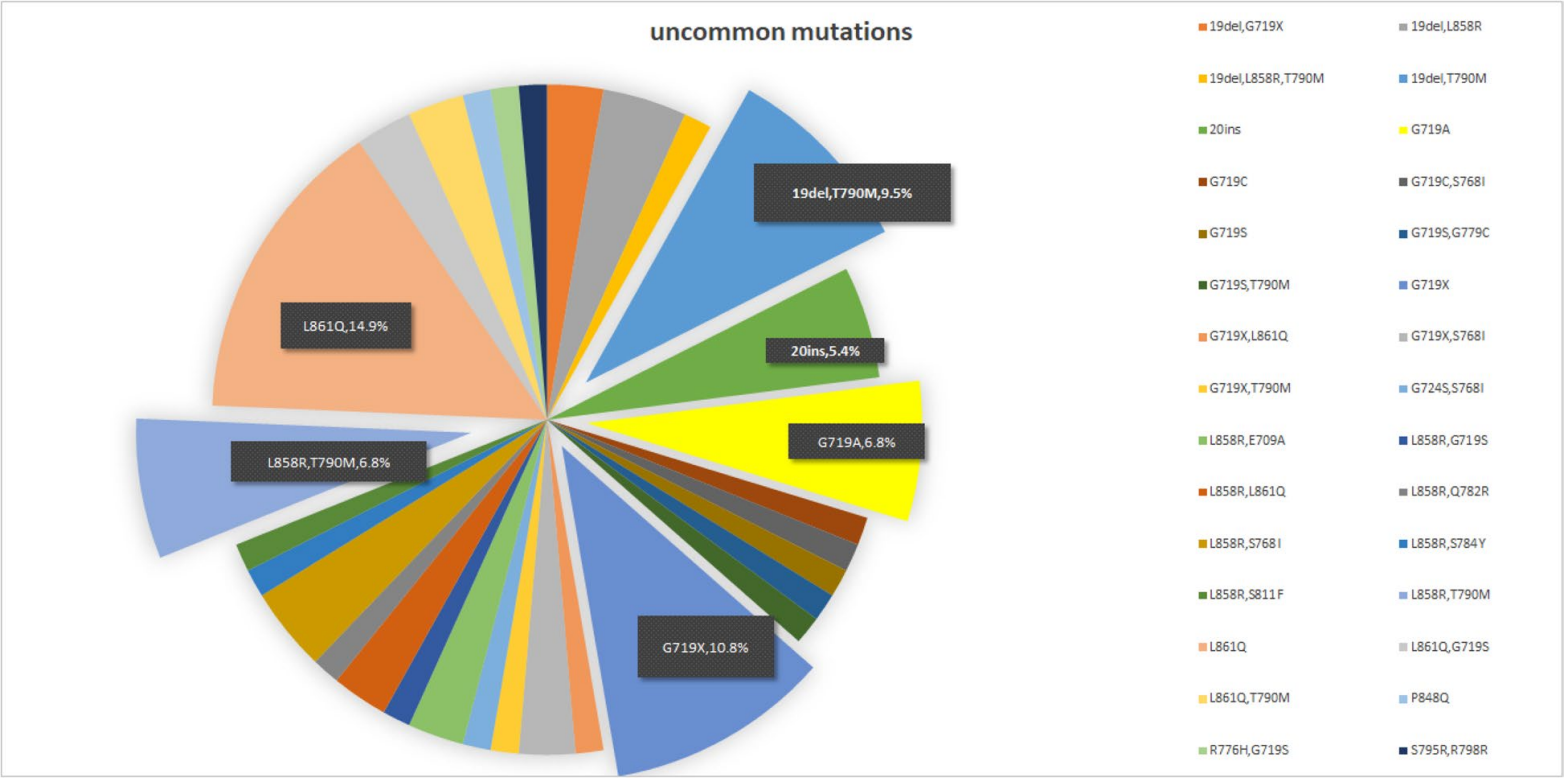
Distribution of uncommon EGFR mutation subtypes. The most common subtypes of uncommon mutations are L861Q (14.9%), G719X (10.8%), 19del/de novo T790M (9.5%), L858R/de novo T790M (6.8%) and G719A (6.8%)

A total of 1081 patients were identified, 74 (6.8%) of whom harbored uncommon mutations in this study. Of the 74 patients, L861Q (14.9%), G719X (10.8%), 19del/de novo T790M (9.5%), L858R/de novo T790M (6.8%) and G719A (6.8%) are the most frequent subtypes of uncommon EGFR mutations. Of note, a total of 17 patients harbored de novo T790M coexisting with or without other subtypes. The detailed distribution of subtypes of uncommon EGFR mutations is summarized in Fig. 2 (and Table S1).

The baseline characteristics of patients in the three groups (uncommon EGFR mutations vs 19del vs L858R) were generally similar, except that baseline bone metastasis developed less frequently in patients with uncommon EGFR mutations (31.1%), when compared with those harboring 19del (43.8%) or L858R mutation (48.0%) (*p* = 0.02). First-generation EGFR-TKIs (including gefitinib and erlotinib), afatinib and Osimertinib, were administered in 86.0, 4.8 and 9.2% of patients in the whole population, respectively. Detail information about baseline clinical features and first-line treatment in each subgroup of patients are summarized in Table 1.

**Table 1:** Baseline characteristics and first-line treatment

	Uncommon (*N* = 74)	19del (*N* = 530)	L858R (*N* = 477)	*p*-value
Sex				0.38
female	41(55.4%)	304(57.4%)	292(61.2%)	
male	33(44.6%)	226(42.6%)	185(38.8%)	
Age, years				0.07
≤ 60	25(33.8%)	228(43.0%)	175(36.7%)	
> 60	49(66.2%)	302(57.0%)	302(60.4%)	
KPS				0.11
90-100	66(89.2%)	411(77.5%)	391(82.0%)	
70-89	7(9.5%)	102(19.2%)	71(14.9%)	
≤ 70	1(1.4%)	17(3.2%)	15(3.1%)	
Lung M				0.18
no	31(41.9%)	233(44.0%)	235(49.3%)	
yes	43(58.1%)	297(56.0%)	242(50.7%)	
Adrenal gland M				0.56
no	72(97.3%)	503(94.9%)	450(94.3%)	
yes	2(2.7%)	27(5.1%)	27(5.7%)	
Liver M				0.90
no	68(91.9%)	494(93.2%)	445(93.3%)	
yes	6(8.1%)	36(6.8%)	32(6.7%)	
Brain M				0.51
no	51(68.9%)	382(72.1%)	355(74.4%)	
yes	23(31.1%)	148(27.9%)	122(25.6%)	
Distant LN M				0.27
no	41(55.4%)	259(48.9%)	255(53.5%)	
yes	33(44.6%)	271(51.1%)	222(46.5%)	
Bone M				0.02
no	51(68.9%)	298(56.2%)	248(52.0%)	
yes	23(31.1%)	232(43.8%)	229(48.0%)	
Oligo-M				0.43
no	64(86.5%)	445(84.0%)	389(81.6%)	
yes	10(13.7%)	84(16.0%)	88(18.4%)	
TKI generations				< 0.001
1st	45(60.8%)	476(89.8%)	409(85.7%)	
2nd	16(21.6%)	20(3.8%)	16(3.4%)	
3rd	13(17.6%)	34(6.4%)	52(10.9%)	

*Abbreviation*: *M* metastasis, *LN* lymph node

### Survival outcomes in the whole population

**Fig. 3:**
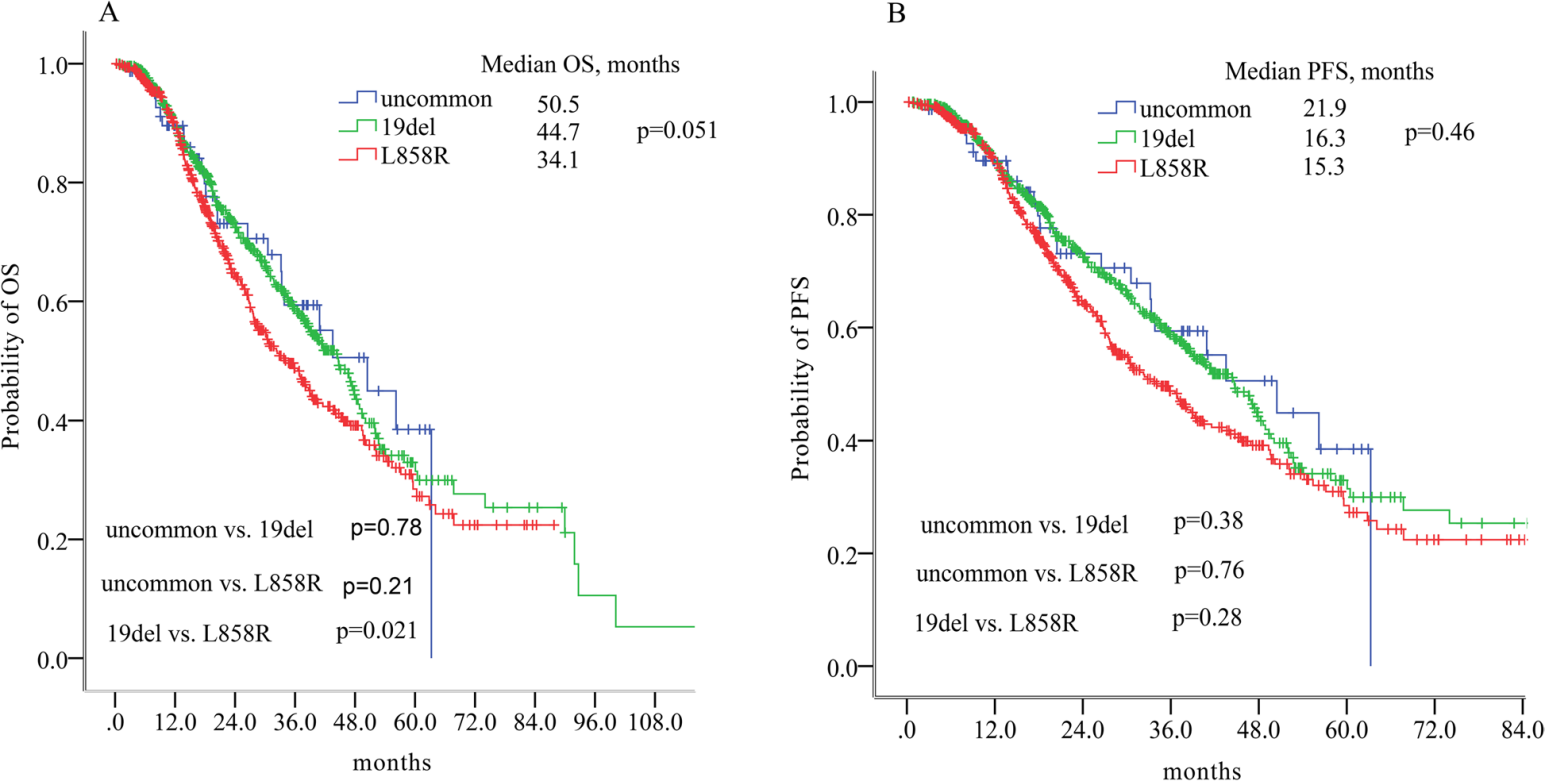
Survival outcomes stratified by EGFR mutation subtypes. **A** Overall survival difference had been seen between patients with 19del and with L858R, but not be seen between patients with uncommon and with 19del or L858R. **B** There was no differences between patients with uncommon and with 19del or L858R in terms of progression free survival

With a median follow-up of 35.0 months (range, 0.3-119.9 months), 619 of 1081patients (57.3%) had developed disease progression. The median progression-free survival (PFS) for all patients was 16.2 months (95%CI = 14.5-17.9 months) and the 1-, 3-, 5-year PFS rate were 61.4, 27.6 and 20.0%, respectively. There was no significant difference in terms of median PFS among patients harboring uncommon mutations, 19del or L858R (21.9 vs 16.3 vs 15.3 months, *p* = 0.46, Fig. 3; and Fig. S1). By the time of data cut-off, 438 patients died. The median OS of all patients was 39.5 months (95%CI = 35.7-43.3 months) and the 1-, 3-, 5-year OS rate were 89.4, 54.3, 31.2%, respectively. There was also no significant difference of OS among the three groups (uncommon mutations vs. 19del vs. L858R, 50.5 vs. 44.7 vs. 34.1 months, *p* = 0.051, Fig. 3; and Fig. S1).

We further compared the differences of survival outcomes between patients harboring distinct uncommon mutation subtypes (*n* ≥ 5) and patients with common mutations (19del or L858R mutations). No significant survival differences between patients with L861Q, G719X, de novo T790M or G719A, and patients with common mutations were found (Fig. S2, S3 and S4) after PSM. Detailed data about survival outcomes of patients with different uncommon mutation subtypes were summarized in supplementary Table S2.

### Survival outcomes in patients with baseline brain metastasis

In our study, 875 (80.9%) patients had baseline brain MRI, which detected 293 patients with baseline BMs. Among these 293 patients, 23(31.1%) harbored uncommon EGFR mutations, 148(27.9%) with 19del and 122(25.6%) with L858R mutation. The median PFS of these 293 patients was 13.7 months (95%CI = 11.6-15.8 months) and median OS was 40.6 months (95%CI = 30.2-50.9 months). There were no significant differences of PFS or OS among patients with baseline BMs in the three groups.

**Fig. 4:**
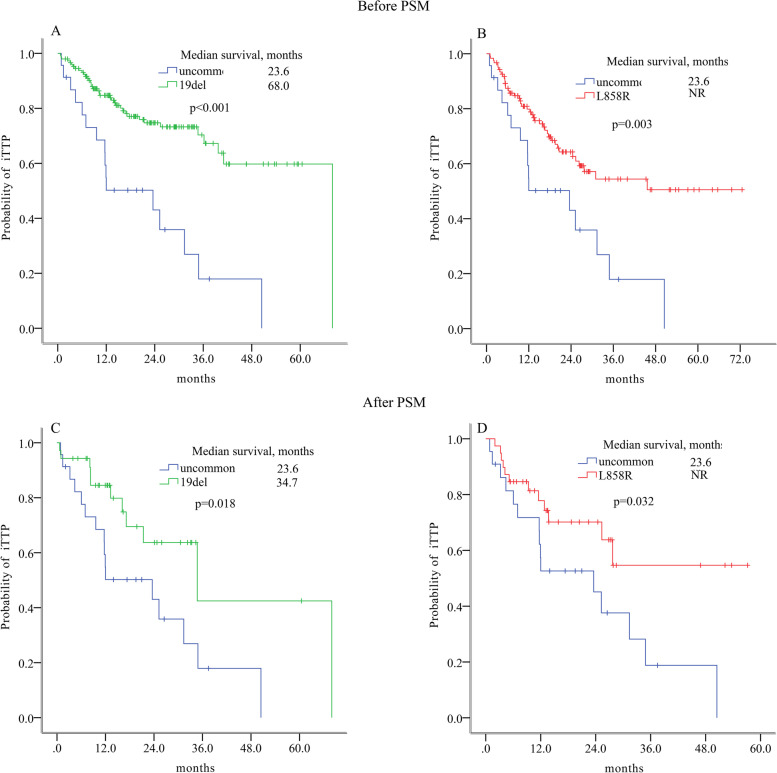
Intracranial time to progression (iTTP) stratified by EGFR mutation subtypes. iTTP of patients with uncommon mutations and those with 19del (**A**) and those with L858R(**B**), before PSM; iTTP of patients with uncommon mutations and those with 19del (**C**) and those with L858R(**D**), after PSM

However, patients with uncommon EGFR mutations had significantly shorter iTTP than those with 19del (*p* < 0.001; Fig. 4A) or L858R (*p* = 0.003; Fig. 4B). After PSM accounting for unbalanced factors, including KPS score, EGFR-TKI generations and baseline bone metastasis, the iTTP of patients with common EGFR mutations was still longer than that of uncommon mutations (uncommon vs. 19del, 23.6 months vs. 34.7 months, *p* = 0.018; uncommon vs. L858R, 23.6 months vs. NR, *p* = 0.032; Fig. 4C-D).

### Survival outcomes in patients with de novo T790M

The median PFS of the 17 patients with de novo T790M mutation was 14.3 months and median OS had not been reached. It did not show any difference of PFS or OS between these 17 patients and those harboring common EGFR mutations before and after PSM as shown in Fig. S5. Of note, 9 out of the 17 patients received Osimertinib during their disease course and these 9 patients tended to have a longer OS than the other 8 patients who did not receive Osimertinib (NR vs. 13.8 months, *p* = 0.08). The details of clinic-pathological characteristics, treatment modalities and survival outcomes of patients with de novo T790M mutation are provided in Table S3 in the supplementary appendix.

### Pattern of treatment failure

**Fig. 5:**
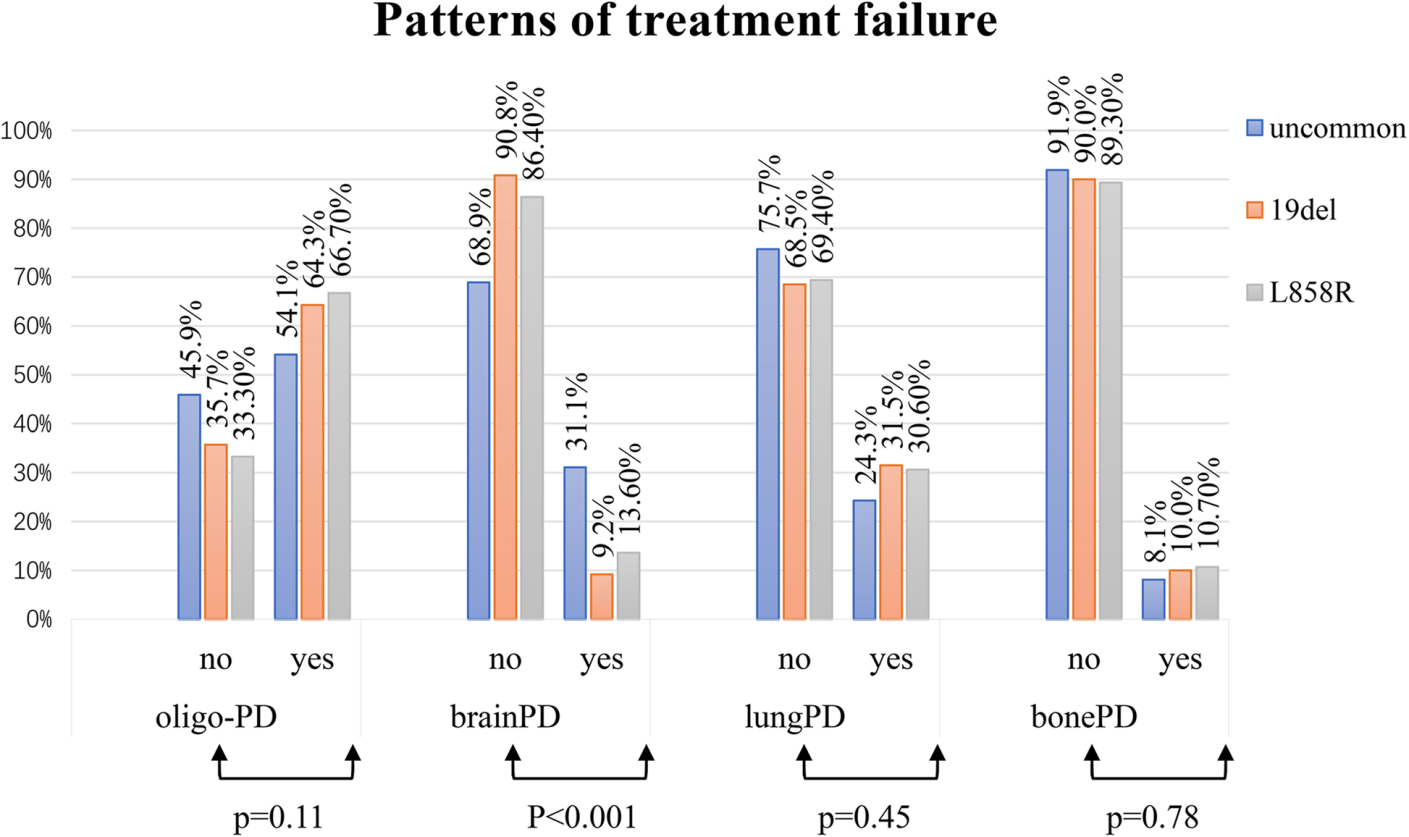
Patterns of treatment failure. The distribution of tumor sites with initial progressive disease after first-line EGFR-TKIs treatment failure were generally similar among the three groups, except for brain progression

As shown in Fig. 5, the distribution of tumor sites with initial progressive disease after first-line EGFR-TKIs treatment failure were generally similar among the three groups, except that there were more patients with cranial PD in the uncommon EGFR mutation group (31.1%) than that in the 19del group (9.2%) or L858R group (13.6%) (*p* < 0.001). Of note, the percentage of patients developing oligo-progressive disease was similar between the three groups (*p* = 0.11).

## Discussion

The clinical outcomes of advanced EGFR-mutant NSCLC patients receiving first-line EGFR-TKIs may be significantly impacted by the subtypes of EGFR mutations [3, 8, 11, 24]. Our study provided clinically relevant information about the baseline characteristics, survival outcomes and patterns of treatment failure of a relatively large number of metastatic NSCLC patients treated with first-line EGFR-TKIs and harboring various types of EGFR mutations. We found a non-inferior survival outcome in patients with rare EGFR mutations than that of the patients with common mutations, except which patients with uncommon EGFR mutations seemed to have a shorter iTTP among those with baseline BM. Also, patients with uncommon EGFR mutations had a higher risk of developing cranial PD, indicating extra treatment modalities may be needed to help prevent and control BMs among these patients. Moreover, Osimertinib-treated patients with de novo T790M mutation were associated with a promising survival, which warranted future validation.

The efficacy of EGFR-TKIs as first-line treatment among patients harboring uncommon EGFR mutations is still under debate and our study indicated that the majority patients could derive clinical benefit from EGFR-TKIs, especially from Osimertinib, except for those with exon 20 insertions (20ins). The incidence of uncommon mutations in this study was 6.8%, which is consistent with previous studies, ranging from 2 to 25% [7, 11, 18, 24–26]. Studies showed that patients with uncommon mutations had inferior response and survival outcomes when treated with EGFR-TKIs, when compared with patients who harbored common mutations [24, 26, 27]. However, the OS and PFS of patients with uncommon mutations in this study were not inferior to that of patients with common mutations. This could be inconsistent with previous studies [10, 24]. The major reason for this inconsistency, in our opinion, was the heterogeneity of EGFR mutation subtypes and EGFR-TKI generations. Firstly, it has been shown that different subtypes of uncommon EGFR mutations may have distinct response to the same EGFR-TKI, while the composition and percentage of EGFR mutation subtypes in each study varied a lot [26, 28]. It was repeatedly shown that patients with 20ins responded poorly to EGFR-TKIs [7, 28–30]. The percentage of patients harboring 20ins was as high as 26.9-31.5% in the previous studies [10, 23] that showed unsatisfactory efficacy results, while the frequency of 20ins mutation in our study was only 5.4% (4/74). Secondly, the percentage of patients with de novo T790M mutation in the previous studies could reach as high as 18.7% (14/75) [3] to 23.7% (9/38) [26]. However, the previous studies investigating the efficacy of EGFR-TKIs treatment among patients harboring uncommon mutations generally included only first- or second-generation EGFR-TKIs, which were generally associated with limited efficacy against de novo T790M mutation [3, 17, 18]. Nevertheless, the majority of patients (9/17) with de novo T790M mutation received Osimertinib treatment in this study.

BM is the most concern for patients with metastatic NSCLC, while little attention has been paid on the clinical outcomes of patients harboring uncommon mutations and with baseline BMs. One former study revealed that patients with BMs and uncommon mutations seemed to respond well to Afatinib [31]. However, there were only 3 patients with BMs in that study. Additionally, a multi-center phase II trial from Korea showed that the intracranial ORR of Osimertinib-treated patients harboring uncommon EGFR mutations was 40% (2/5) [15]. Nevertheless, the survival benefit was limited in the Korea study, with a median iTTP shorter than 9 months [32]. The iTTP of patients with uncommon mutations was also significantly shorter than that of patients with common mutations in our study. Meanwhile, even there were seventeen (24.3%) of 74 patients with rare mutation received Osimertinib treatment during their disease course, there were still higher percentage of patients with uncommon EGFR mutations developed their initial disease progression in the brain than those with common mutations (31.1% vs 9.2, 13.6%, *p* < 0.001). Similar phenomenon had been reported in a previous study [32]. Taken together, EGFR-TKIs alone may be less effective against BMs in patients harboring uncommon EGFR mutations, and extra treatment modalities may be needed.

Local therapy, such as stereotactic radiosurgery (SRS), whole brain radiotherapy (WBRT) and surgery, may be the choices of the extra treatment modalities for patients harboring uncommon mutations and BMs, which have been proved to play an important role in the treatment of BMs in NSCLC patients who harbor common mutations. Previous studies reported that patients with EGFR-mutant NSCLC and limited numbers of BMs seemed to obtain survival benefit from upfront cranial local therapy [33, 34]. Lee et al. showed that proper cranial local therapy could improve OS even in the era of Osimertinib treatment [35], which was consistent with our previous study [36]. However, there was little information that explored the effectiveness of cranial local therapy in the management of BMs form patients harboring uncommon EGFR mutations. There was one case report [37] presenting that one patient with exon18 G719S mutation and BMs obtained a 49 months of intracranial PFS and 67 months of OS after receiving combined brain radiotherapy and erlotinib treatment. The clinical value and optimal timing of cranial local therapy against BMs from NSCLC patients harboring uncommon EGFR mutations deserved further investigation.

The exact frequency of de novo T790M mutation and its sensitivity to common EGFR-TKIs remain controversial [12, 16]. The post-hoc analysis of LUX-Lung 2, LUX-Lung 3 and LUX-Lung 6 showed that patients with de novo T790M could benefit little from Afatinib [3]. Another study including nine patients showed that patients with 19del or L858R combined with T790M were less sensitive to first-generation of EGFR-TKIs [7]. Evidence from many retrospective studies showed that the presence of de novo T790M may mean poor prognosis [16, 17, 38, 39]. And patients with higher T790M relative allele frequency had worse prognosis [40], one patient who received Osimertinib treatment after first-generation EFGR-TKI failure gained a partial response and a sustained response. Osimertinib may be an effective solution for patients with de novo T790M mutation. Meanwhile, Zhang et al. reported that patients with de novo T790M could benefit longer survival from Osimertinib than that from first generation EGFR-TKIs [19]. However, patients included in that study were treated with different lines. In our study, most of the patients with de novo T790M mutation received Osimertinib treatment, which induced promising survival results. Several studies have found that tumors with acquired T790M mutation generally had more indolent biological behavior and patients with acquired T790M mutation had more favorable prognosis, when compared with those without T790M mutation [41–43]. Thus, de novo T790M mutation may define a special subset of EGFR-mutant NSCLC with favorable prognosis when treated with first-line Osimertinib, which warrants further validation with a large sample size study.

This study has some inherent limitations. First, the retrospective nature of this study makes it impossible to collect completed data about treatment toxicities and acquired resistance mechanisms for each patient. Second, the number of patients in the uncommon EGFR mutation group was limited, so that the conclusions in our study need to be interpreted with caution. Third, there were only a minority of patients in the uncommon mutation group received second-generation EGFR-TKIs treatment. Afatinib was approved in China after 2017 when it was not covered by the national medical insurance system, which made it inaccessible to the majority of patients in our study. However, despite the above drawbacks, our study provided clinically relevant information about treatment outcomes of first-line EGFR-TKI in patients with different EGFR subtypes and various baseline clinical features, especially those with BMs and/or de novo T790M mutation.

## Conclusions

In conclusion, first-line EGFR-TKIs treatment was generally associated with similar survival outcomes in advanced EGFR-mutant NSCLC patients with common and uncommon mutations, except that it seemed less effective treating and preventing BMs in patients harboring uncommon mutations. Meanwhile, Osimertinib treatment was associated with promising survival outcomes in patients with de novo T790M mutation, which warranted further validation.

## Supplementary Information

**Additional file 1: Figure S1.** Survival outcomes before and after PSM (accounting for TKI generation, brain M and oligo-M) between patients with uncommon mutations and patients harboring 19del or L858R.

**Additional file 2: Figure S2.** Survival outcomes before and after PSM (accounting for TKI generation, brain M and oligo-M) between patients with L861Q mutations and patients harboring 19del or L858R.

**Additional file 3: Figure S3.** Survival outcomes before and after PSM (accounting for bone M, lung M, brain M and oligo-M) between patients with G719X mutations and patients harboring 19del or L858R.

**Additional file 4: Figure S4.** Survival outcomes before and after PSM (accounting for TKI generation, gender, brain M and oligo-M) between patients with G719A mutations and patients harboring 19del or L858R.

**Additional file 5: Figure S5.** Survival outcomes before and after PSM (accounting for TKI generation, brain M and oligo-M) between patients with T790M and patients harboring 19del or L858R.

**Additional file 6: Table S1.** The detail of uncommon mutations. **Table S2.** Survival outcome of several rare mutations. **Table S3.** Details of the 17 patients with de novo T790M mutations.

## Data Availability

All data generated or analysed during this study are included in this published article.

## Abbreviations

NSCLC: Non-small cell lung cancer
TKIs: Tyrosine kinases inhibitor
EGFR: Epidermal growth factor receptor
ORR: Objective response rate
CT: Chest computed tomography
MRI: Brain magnetic resonance imaging
PET-CT: Positron Emission Tomography
Ittp: Intracranial time to progression
PD: Progressive disease
SRS: Stereotactic radiosurgery
