# Supplementary material for: Clinical outcomes of advanced non-small cell lung cancer patients harboring distinct subtypes of EGFR mutations and receiving first-line tyrosine kinase inhibitors: brain metastasis and de novo T790M matters
Source: BMC Cancer. 2022 Feb 21;22:198. doi: 10.1186/s12885-022-09245-5 (PMC8862369; doi:10.1186/s12885-022-09245-5)
Supplement: Supplementary file 6 — Additional file 6: Table S1. The detail of uncommon mutations. Table S2. Survival outcome of several rare mutations. Table S3. Details of the 17 patients with de novo T790M mutations. [file 12885_2022_9245_MOESM6_ESM.docx]

**Table S1** The detail of uncommon mutations.

| Uncommon mutations | frequency |
| --- | --- |
| L861Q | 11(14.9%) |
| G719X | 8(10.8%) |
| 19del, T790M | 7(9.5%) |
| G719A | 5(6.8%) |
| L858R, T790M | 5(6.8%) |
| 20ins | 4(5.4%) |
| 19del, L858R | 3(4.1%) |
| L858R, S768I | 3(4.1%) |
| G719X,S768I | 2(2.7%) |
| L858R, E709A | 2(2.7%) |
| L858R, L861Q | 2(2.7%) |
| L861Q, G719S | 2(2.7%) |
| L861Q, T790M | 2(2.7%) |
| 19del, G719X | 2(2.7%) |
| 19del, L858R, T790M | 1(1.4%) |
| G719C | 1(1.4%) |
| G719C, S768I | 1(1.4%) |
| G719S | 1(1.4%) |
| G719S, G779C | 1(1.4%) |
| G719S, T790M | 1(1.4%) |
| G719X, L861Q | 1(1.4%) |
| G719X, T790M | 1(1.4%) |
| G724S, S768I | 1(1.4%) |
| L858R, G719S | 1(1.4%) |
| L858R, Q782R | 1(1.4%) |
| L858R, S784Y | 1(1.4%) |
| L858R, S811F | 1(1.4%) |
| P848Q | 1(1.4%) |
| R776H, G719S | 1(1.4%) |
| S795R, R798R | 1(1.4%) |

**Table** **S2** Survival outcome of several rare mutations.

|  | n | PFS, months | OS, months |
| --- | --- | --- | --- |
| G719A | 5 | 6.2 | 17.9 |
| G719X | 8 | 14.1 | 33.8 |
| L861Q | 11 | 9.9 | 56.2 |
| T790M | 17 | 14.3 | NR |

**Table S3** Details of the 17 patients with de novo T790M mutations.

| Patient  No. | age | gender | EGFR mutations | Baseline  BM | Baseline  OMD^※^ | 1^st^-line  TKI | Best response | PFS  (m^∈^) | OPD^＆^ | brain PD | OS  (m) |
| --- | --- | --- | --- | --- | --- | --- | --- | --- | --- | --- | --- |
| 1 | 44 | F | L861Q, T790M | Yes | No | Gefitinib | SD | 9.1 | Yes | No | 17.3 |
| 2 | 82 | F | 19del, T790M | Yes | Yes | Osimertinib | SD | 21^+^ | \ | \ | 21^+^ |
| 3 | 66 | F | 19del, T790M | Yes | No | Osimertinib | PR | 23.8 | No | No | 37.5^+^ |
| 4 | 59 | M | G719X, T790M | No | Yes | Gefitinib | PR | 21.9 | Yes | Yes | 58.7^+^ |
| 5 | 49 | F | 19del, T790M | No | No | Osimertinib | SD | 16.8^+^ | \ | \ | 16.8^+^ |
| 6 | 65 | F | 19del, T790M | Yes | No | Osimertinib | PR | 10.0 | Yes | No | 19.5^+^ |
| 7 | 50 | F | L858R, T790M | No | No | Osimertinib | PR | 29.1 | Yes | No | 31.4^+^ |
| 8 | 37 | F | 19del, L858R,  T790M | No | No | Gefitinib | PD | 1.0 | No | No | 7.4 |
| 9 | 71 | F | L858R, T790M | Yes | No | Gefitinib | PR | 1.4 | Yes | Yes | 9.4 |
| 10 | 65 | M | G719S, T790M | No | Yes | Gefitinib | PR | 29.7^+^ | \ | \ | 29.7^+^ |
| 11 | 58 | F | 19del, T790M | No | No | Osimertinib | PR | 48.8^+^ | \ | \ | 48.8^+^ |
| 12 | 64 | F | 19del, T790M | No | No | Gefitinib | SD | 4.4^+^ | \ | \ | 4.4^+^ |
| 13 | 31 | M | L858R, T790M | No | No | Osimertinib | PR | 14.3^+^ | \ | \ | 14.3^+^ |
| 14 | 61 | F | L858R, T790M | No | No | Erlotinib | PD | 1.7 | No | Yes | 13.8 |
| 15 | 71 | M | L861Q, T790M | No | No | Osimertinib | PD | 3.4 | Yes | No | 3.4^+^ |
| 16 | 53 | F | 19del, T790M | No | No | Erlotinib | SD | 11.0 | Yes | No | 46.6 |
| 17 | 47 | M | L858R,T790M | No | No | Osimertinib | PD | 2.1 | No | No | 3^+^ |

^※^OMD，oligo-metastasis disease; ^∈^m, months; ^＆^OPD, oligo-progression disease.
